# Perception and the influence of information toward e-cigarette smoking behavior

**DOI:** 10.18332/tid/189396

**Published:** 2024-06-20

**Authors:** Pantira Parinyarux, Panida Ditsawanon, Athichar Chanwuthinun, Adinat Umnuaypornlert, Surasak Saokaew, Preechaya Tajai

**Affiliations:** 1 Social and Administrative Pharmacy Department, Faculty of Pharmaceutical Sciences, Chulalongkorn University, Bangkok, Thailand; 2 Faculty of Pharmacy, Payap University, Chiang Mai, Thailand; 3 Faculty of Pharmacy, Chiang Mai University, Chiang Mai, Thailand; 4 School of Pharmaceutical Sciences, University of Phayao, Phayao, Thailand; 5 Department of Forensic Medicine, Faculty of Medicine, Chiang Mai University, Chiang Mai, Thailand

**Keywords:** electronic cigarette, perception, information, smoking behavior, vaping

## Abstract

**INTRODUCTION:**

Perceptions, personal perspectives, and public awareness of e-cigarette information have a significant impact on e-cigarette smoking behavior, and provide comprehensive information that can help reduce interest in e-cigarette smoking and reduce the number of new smokers. This study aimed to investigate the perceptions towards e-cigarettes related to e-cigarette use and how that information related to people’s use of them.

**METHODS:**

The data for this cross-sectional study were collected via an online questionnaire. Thai nationals who were aged ≥18 years provided data between June 2021 and January 2022. Multivariable logistic regression and the chi-squared test were used to analyze the data.

**RESULTS:**

There were 340 respondents, 76 e-cigarette users, and 264 non-e-cigarette users. Most of the perceptions of information that differed statistically significantly between e-cigarette users and non-e-cigarette users included information on regulations, products, health effects, and the effectiveness of smoking cessation. The association between factors and e-cigarette smoking behavior revealed that the perception of the product information and male gender were associated with e-cigarette smoking behavior (AOR=13.59; 95% CI: 2.35–78.60, and AOR=5.19; 95% CI: 2.87–9.40, respectively).

**CONCLUSIONS:**

The perception of e-cigarette product information and male gender were associated with e-cigarette smoking behavior.

## INTRODUCTION

The global prevalence of electronic cigarettes or e-cigarette use is 11%, which tends to increase continuously. However, they are illegal tobacco products in more than 25 countries around the world, including Thailand^1,2^. In Thailand, the number of e-cigarette smokers is likely to increase from year to year. In the years since 2015, the number of e-cigarette smokers has increased by as much as five times^3^.

E-cigarettes are devices that convey nicotine. With varying concentrations of nicotine reagents packed in small cartridges, smokers receive liquid nicotine through thermal vaporization without burning tobacco leaves^4^. There are misconceptions about the risks and benefits of e-cigarettes. The data currently show that e-cigarettes are less harmful and less carcinogenic than smoking ordinary cigarettes^5,6^. However, it cannot be concluded that e-cigarettes do not pose a health hazard, as substances that affect the cardiovascular system have also been identified^5^. There is also a lack of academic confirmation of the long-term impact on health and safety^5,7^. In addition, the current public perception of e-cigarettes lacks credibility, especially in terms of the efficacy of smoking cessation supports^5^. The data are also inconsistent across multiple studies. Numerous studies have shown that e-cigarettes are more effective in helping smokers quit smoking than nicotine replacement therapy^8-12^. However, certain studies indicate that the users of e-cigarettes are just as likely to quit smoking as those who do not use e-cigarettes^13,14^. In addition, a review of clinical studies concluded that data on the efficacy of cessation-assisted e-cigarettes are limited and that long-term safety outcomes are unknown^15^.

In Thailand, a large amount of information is available on the spread and sale of e-cigarette products on the black market or online^16^. E-cigarette dealers and those who supported e-cigarettes in Thailand sought to release only positive information to persuade and encourage the use of e-cigarettes. This may have had an impact on the perception and belief of new smokers, making them interested in experiencing smoking^17,18^. According to studies on smoking habits in Thailand, it has been reported that the factors and conditions for initiating e-cigarette use were, in part, due to advertising. Most smokers searched for information on e-cigarettes before attempting to smoke, and the sources searched were online media and inquiries from sellers, including those they trusted to provide information^19^. Therefore, people could easily access information about e-cigarettes. A study in the United States also found that the general public perceived information about e-cigarettes through television, media, and word-of-mouth. Most were related to information on the health effects of e-cigarettes and the efficacy of quitting smoking or reducing the number of smoking regular cigarettes^20^.

The way people view e-cigarettes is based on their perspective, which in turn affects how they respond and behave^21^. It is essential to understand how aware the public is about e-cigarette information in order to comprehend their interest in e-cigarette products. Studies have shown that the way e-cigarettes are advertised has an impact on people’s e-cigarette smoking behavior. As a result, studies have indicated experimentation with e-cigarette products and belief that e-cigarettes are less harmful than regular cigarettes^22-24^. Accordingly, to control the spread of e-cigarettes, providing the public with multi-dimensional information about e-cigarettes to gain specific awareness, could help reduce interest in e-cigarette smoking, potentially reducing the number of new smokers.

This study aimed to investigate the perception of information about e-cigarettes and the relationship of information that influenced e-cigarette smoking behavior. This would be considered important information in promoting policies regulating tobacco consumption in Thailand.

## METHODS

### Population and sample groups

This research was a cross-sectional study using an online questionnaire. Data collection was conducted from June 2021 to January 2022, following the approval of the Human Experimentation Committee Research Institute for Health Sciences (RIHES), Chiang Mai University, Chiang Mai, Thailand (No. 31/2021).

The population consisted of people of Thai nationality who were aged ≥18 years. The sample size was calculated from a previous study in which 16.1% of participants reported having smoked electronic cigarettes, most of whom were aware of them^25^. At least 208 people were to be included in a survey using the STATA command *svysampsi* with a power of 80% and a significance level of 0.05. Three hundred fifty-seven questionnaires were received, and 17 were excluded due to incompleteness. Three hundred forty effective questionnaires were included in the analysis through this survey.

### Data collection methods

The survey data were collected online using Google Forms. Thai citizens aged ≥18 years were requested to complete the online questionnaire. Researchers promoted the link and QR code of questionnaires through social networks chosen by Thai citizens: Facebook, LINE, and Twitter^26^.

### Research instruments

This study was based on a questionnaire developed from data from the review literature. The questionnaire was assessed for content validity and language clarity by three experts based on an item-objective congruence index of at least 0.7. This questionnaire was subsequently tested in a target group of 30 people. It was then developed to meet the criteria for reliability, language clarity, and acceptable appropriateness. The questionnaire in this research had an alpha Cronbach coefficient of 0.92. It consisted of three sections: 1) general information about respondents, all samples were gathered for general data, including age, gender, region, education level, and occupation. Data on users of e-cigarettes was gathered, including information on their use of the devices, their levels of nicotine addiction as determined by the Heaviness of Smoking Index (HSI), and their intentions or plans to stop smoking; 2) experience in accessing data on e-cigarette information, selective response using five Likert scales; 3) questions about the perception of e-cigarette information. The answers were chosen from three rating scales. In section 3, there were also questions about perceptions of each domain in order to determine the relationship between data perception and e-cigarette smoking behavior.

### Data analysis

Quantitative data were analyzed with the Statistical Package for Social Science and interpreted with descriptive statistics, including frequency, percentage, mean, and standard deviation. The chi-squared test was used as the first inferential statistical method (if an expected cell count of less than five equals or greater than 20%, Fisher’s exact test was used instead). The independent t-test was used to find the difference in the general data between the sample groups that used and did not use e-cigarettes. The experiences in accessing and perceiving e-cigarette information on each side were compared between these two groups using the chi-squared test. All tests were 2-tailed and assumed significance at p<0.05. The demographic data and perceiving e-cigarette information as a function of various predictors in the data set were evaluated using univariable logistic regression, and those results were expressed as odds ratios (ORs) and 95% CIs. The characteristic variables with p<0.05 in univariable logistic regression and those identified by previous studies^27,28^ and perceiving e-cigarette information as predictors of e-cigarette smoking behavior, were submitted to multivariable logistic regression (enter method) to establish their independent association with e-cigarette smoking behavior while statistically controlling for other factors. Adjusted odds ratios (AORs) and 95% confidence intervals (CIs) were used to express the results.

## RESULTS

This study included 340 respondents, 76 e-cigarette users (22.4%) and 264 non-e-cigarette users (77.6%). Age, gender, residence, and job were statistically different between the two groups, according to their characteristics data (p<0.05). The majority of e-cigarette users were men, and they were typically younger than non-e-cigarette users (Table 1).

**Table 1:** Demographic characteristics of e-cigarette and non-e-cigarettes users, aged ≥18 years, Thailand, June 2021 – January 2022 (N=340)

*Characteristics*	*Total (N=340)*	*E-cigarette users (N=76)*		*Non-e-cigarette users (N=264)*		p*
	*n (%)*	*n*	*%*	*n*	*%*	
**Age** (years), mean ± SD	34.1 ± 13.20	31.2 ± 8.33	34.9 ± 14.21	0.005		
**Gender**						<0.001
Male	138 (40.6)	54	71.1	84	31.8	
Female	202 (59.4)	22	28.9	180	68.2	
**Region**						0.005
Central	123 (36.2)	37	48.7	86	32.6	
Northern	165 (48.5)	26	34.2	139	52.7	
Northeastern/Eastern	20 (5.9)	5	6.6	27	10.2	
Southern/Western	32 (9.4)	8	10.5	12	4.5	
**Education**						0.135
Lower than BA	35 (10.3)	6	7.9	29	11.0	
Bachelor’s	213 (62.6)	56	73.7	157	59.5	
Higher than BA	89 (26.2)	14	18.4	75	28.4	
Other	3 (0.9)	0	0.0	3	1.1	
**Occupation**						<0.001
University student	112 (32.9)	23	30.3	89	33.7	
Officer	75 (22.1)	21	27.6	54	20.5	
Private businessman	28 (8.2)	16	21.1	12	4.5	
Civil servant	51 (15.0)	10	13.2	41	15.5	
Freelance/Employee	21 (6.2)	4	5.3	17	6.4	
Unemployed	11 (3.2)	2	2.6	9	3.4	
Other	42 (12.4)	0	0.0	42	15.9	

* The p-values were determined by independent t-test or chi-squared test, significance at p<0.05.

Of the 76 e-cigarette users, 64.0% continued to consume cigarettes regularly. They consumed other tobacco products (75.0%) as well. To quit smoking was their primary reason for using e-cigarettes (53.7%). The majority of respondents (85.5%) had low levels of nicotine addiction, according to the HSI (Table 2).

**Table 2:** Characteristics and general information of e-cigarette users consuming e-cigarettes, aged ≥18 years, Thailand, June 2021 – January 2022 (N=76)

*Characteristics*	*E-cigarette users*	
	*n*	*%*
**Whether or not to smoke tobacco products** (N=75)*		
Still smoke e-cigarette (current e-cigarette smokers)	48	64.0
Quit smoking e-cigarette for over a month (ex-smokers)	27	36.0
**Length of smoking e-cigarette**s (months)*, mean ± SD	10.4 ± 11.77	
**Use of other tobacco products**		
Yes	57	75.0
No	19	25.0
**Purpose of using e-cigarettes** (N=67)*		
To quit smoking traditional cigarettes	36	53.7
To alternate with traditional cigarettes	6	9.0
To experiment	18	26.9
Other	7	10.4
**Levels of nicotine addiction, as measured by HSI** (N=76)		
5–6 (severe)	0	0.0
3–4 (moderate)	11	14.5
0–2 (low)	65	85.5
**Intention or plan to quit smoking among current e-cigarettes users** (N=44)*		
Do not want to quit/smoke e-cigarettes	9	20.5
Want to quit but no date is specified	26	59.1
Want to quit in 6 months	7	15.9
Want to quit in a month	2	4.5
**Receive help or recommendations for e-cigarettes smoking cessation** (N=71)*		
Never receive recommendations	48	67.6
Ever receive recommendations from medical staff	11	15.5
Ever receive recommendations from pharmacists	7	9.9
Ever receive recommendations from friends	1	1.4
Other	4	5.6

* Missing data.

### Channels to access e-cigarette information

The results of our study revealed that e-cigarette users and non-users had statistically significantly different ways of accessing and searching for information about e-cigarettes (p<0.05). E-cigarette users accessed e-cigarette information more frequently than non-e-cigarette users in terms of access. Additionally, they searched for e-cigarette information through various channels more often than non-e-cigarette users did. E-cigarette users preferred to get information on e-cigarettes from people with a smoking history (51.3%) and mostly viewed samples of genuine smokers (64.5%) (Table 3).

**Table 3:** Experiences in accessing e-cigarette information by e-cigarette and non-e-cigarette users, aged ≥18 years, Thailand, June 2021 – January 2022 (N=340)

*Channels to access e-cigarette information*	*E-cigarette users (N=76) n (%)*	*Non-e-cigarette users (N=264) n (%)*	p*
**Obtaining information**			
**Advertisements, web banners on various websites**			0.019
Always-often	13 (17.1)	35 (13.3)	
Sometimes	23 (30.3)	46 (17.4)	
Hardly-never	40 (52.6)	183 (69.3)	
**Advertisements on Facebook, Instagram, social networks/online communities**			<0.001
Always-often	18 (23.7)	41 (15.5)	
Sometimes	27 (35.5)	41 (15.5)	
Hardly-never	31 (40.8)	182 (68.9)	
**Persuasion or recommendations from friends, people around**			<0.001
Always-often	30 (39.5)	23 (8.7)	
Sometimes	25 (32.9)	45 (17.0)	
Hardly-never	21 (27.6)	196 (74.2)	
**Seeing other people smoke**			<0.001
Always-often	49 (64.5)	103 (39)	
Sometimes	16 (21.1)	58 (22.0)	
Hardly-never	11 (14.5)	103 (39.0)	
**Searching for information**			
**Search on the Internet on their own**			<0.001
Always-often	35 (46.1)	19 (7.2)	
Sometimes	19 (25.0)	33 (12.5)	
Hardly-never	22 (28.9)	212 (80.3)	
**Ask people who smoke e-cigarettes directly**			<0.001
Always-often	39 (51.3)	35 (13.3)	
Sometimes	26 (34.2)	36 (13.6)	
Hardly-never	11 (14.5)	193 (73.1)	
**Ask from websites or e-cigarette sales pages**			<0.001
Always-often	25 (32.9)	9 (3.4)	
Sometimes	17 (22.4)	19 (7.2)	
Hardly-never	34 (44.7)	236 (89.4)	

* The p-values were determined by chi-squared test, significance at p<0.05.

### E-cigarette information perception

The survey’s findings revealed that e-cigarette users and non-users had significantly different perceptions of regulation information, product information, health effects information, and the success of quitting smoking. It has also been shown that e-cigarette users have been more knowledgeable about e-cigarettes than non-users (Table 4).

**Table 4:** Perception of e-cigarette information by e-cigarette and non-e-cigarette users, aged ≥18 years, Thailand, June 2021 – January 2022 (N=340)

	*E-cigarette information*	*Data perception*	*E-cigarette users (N=76)*	*Non-e-cigarette users (N=264)*	p*
	*Regulation information*		*n (%)*	*n (%)*	
**R1**	Currently, e-cigarettes are considered a tobacco product in Thailand.	Aware	22 (28.9)	88 (33.3)	0.471
		Not aware/not sure	54 (71.1)	176 (66.7)	
**R2**	Currently, e-cigarettes are considered a prohibited commodity in Thailand.	Aware	59 (77.6)	174 (65.9)	0.052
		Not aware/not sure	17 (22.4)	90 (34.1)	
**R3**	Currently, the import of e-cigarettes, baraku, and electric baraku is prohibited in Thailand.	Aware	62 (81.6)	155 (58.7)	<0.001
		Not aware/not sure	14 (18.4)	109 (41.3)	
**R4**	Currently, e-cigarettes and reagents are prohibited in Thailand.	Aware	59 (77.6)	164 (62.1)	0.012
		Not aware/not sure	17 (22.4)	100 (37.9)	
	***Product information***				
**P1**	Nicotine concentrations in e-cigarette reagents differ. Some reagents lack nicotine.	Aware	57 (75.0)	123 (46.6)	<0.001
		Not aware/not sure	19 (25.0)	141 (53.4)	
**P2**	E-cigarettes heat the vaporization process of e-cigarette reagents.	Aware	69 (90.8)	143 (54.2)	<0.001
		Not aware/not sure	7 (9.2)	121 (45.8)	
**P3**	E-cigarette waste can release heavy metals, such as lead and cadmium.	Aware	31 (40.8)	94 (35.6)	0.409
		Not aware/not sure	45 (59.2)	170 (64.4)	
**P4**	E-cigarette batteries are prone to explosions or fires.	Aware	50 (65.8)	102 (38.6)	<0.001
		Not aware/not sure	26 (34.2)	162 (61.4)	
	***Health effects information***				
**H1**	Nicotine in e-cigarette reagents makes the body addicted to smoking.	Aware	57 (75.0)	165 (62.5)	0.044
		Not aware/not sure	19 (25.0)	99 (37.5)	
**H2**	Nicotine in e-cigarettes leads to lung cancer and respiratory diseases. It affects the cardiovascular system.	Aware	51 (67.1)	177 (67.0)	0.992
		Not aware/not sure	25 (32.9)	87 (3.0)	
**H3**	Propylene glycol in e-cigarettes is a component of vapor formation. When touched or inhaled, the irritation may occur in the oral cavity, throat, lungs, and eyes. It can also cause coughing.	Aware	48 (63.2)	115 (43.6)	0.003
		Not aware/not sure	28 (36.8)	149 (56.4)	
**H4**	Flavoring and tasting agents can cause respiratory and pulmonary problems when they become vapor.	Aware	47 (61.8)	141 (53.4)	0.193
		Not aware/not sure	29 (38.2)	123 (46.6)	
**H5**	Inhaling secondhand vapors from e-cigarette agents will affect the circulatory system and cause cancer in smokers and those around them.	Aware	38 (50.0)	146 (55.3)	0.414
		Not aware/not sure	38 (50.0)	118 (44.7)	
**H6**	Currently, there is no safety data on the long-term use of e-cigarettes.	Aware	52 (68.4)	127 (48.1)	0.002
		Not aware/not sure	24 (31.6)	137 (51.9)	
	***Efficacy of smoking cessation***				
**C1**	There is no evidence confirming that the use of e-cigarettes is effective in helping to stop smoking ordinary cigarettes.	Aware	49 (64.5)	109 (41.3)	<0.001
		Not aware/not sure	27 (35.5)	155 (58.7)	
**C2**	Studies have shown that people who want to quit smoking ordinary cigarettes can do it, no matter what they smoke, without any difference.	Aware	41 (53.9)	96 (36.4)	0.006
		Not aware/not sure	35 (46.1)	168 (63.6)	
**C3**	Studies have shown that people who want to stop smoking ordinary cigarettes can do so without any difference, no matter which e-cigarette reagents they use contain nicotine or not.	Aware	44 (57.9)	91 (34.5)	<0.001
		Not aware/not sure	32 (42.1)	173 (65.5)	

* The p-values were determined by chi-squared test, significance at p<0.05.

### Predictors for e-cigarette smoking behavior: univariable and multivariable logistic regression

For univariable logistic regression, the association between each factor and the e-cigarette smoking behavior was tested. The factors, including gender, age, occupation, perception of regulation, perception of the product, perception of the efficacy of smoking cessation, and perception of health effect information, were tested. Only a few variables, including gender, perception of the product, and perception of the efficacy of smoking cessation, were associated with e-cigarette smoking behavior (all p<0.05) (Table 5). Multivariable logistic regression analyses of perceived cigarette smoking and characteristics variables revealed findings consistent with the sample’s use of e-cigarettes for smoking. The information of the product and male gender was associated with e-cigarette smoking behavior (AOR=13.59; 95% CI: 2.35–78.60 and AOR= 5.19; 95% CI: 2.87–9.40, respectively) (Table 5).

**Table 5:** Multivariable and univariable analysis for predictors of e-cigarette smoking behavior among e-cigarette users and non-e-cigarette users, aged ≥18 years, Thailand, June 2021 – January 2022 (N=340)

*Factors*	*OR*	*95% CI*	p*
**Univariable logistic regression**			
Gender	5.26	3.01–9.20	<0.001
Perception of product information	4.36	1.95–9.75	<0.001
Perception of efficacy of smoking cessation	4.32	1.51–12.33	0.006
	***AOR***	***95% CI***	***p***
Multivariable logistic regression**			
Gender	5.19	2.87–9.40	<0.001
Perception of product information	13.59	2.35–78.60	0.004

* Significance at p<0.05. AOR: adjusted odds ratio.

** Adjusted for age, gender, occupation, perception of regulation, product, efficacy of smoking cessation, and health effect information.

## DISCUSSION

This study investigated the perception of information about e-cigarettes and the relationship of factors that influenced e-cigarette smoking behavior. According to the findings of the study, non-e-cigarette users interpreted e-cigarette information differently from users in terms of regulation, products, health effects, and the efficacy of smoking cessation. The results have shown that the information of product and male gender are associated with e-cigarette smoking behavior.

The majority of e-cigarette users in this study had low levels of nicotine dependence. They desired to use e-cigarettes to try them. The varying concentrations of nicotine found in the e-cigarette reagents raised concerns that this group of smokers might develop higher nicotine addiction levels. It can cause addiction by activation of mesolimbic brain reward circuitry and the release of the neurotransmitter dopamine, which contributes to the development of addiction^29^. E-cigarette users, mostly university students, with low levels of nicotine addiction formed a group with high rates of e-cigarettes and easy access to them^19^.

In this study, e-cigarette users were found to seek and receive more information about e-cigarettes from various channels compared to non-users, primarily through personal experiences and social interactions with smokers in the US. Half of the sample discussed e-cigarettes, with nearly one in three recommending them^20^. This is similar to findings from earlier studies in Thailand, where the presence of e-cigarette role models influenced smoking behavior^19,30^. Despite Thailand’s prohibition of e-cigarettes, over 64.5% of users were influenced by observing others smoking them, proposing a lack of fear of prosecution and belief in mild punishments for violating e-cigarette laws^31^. This aligns with studies indicating that legal perceptions do not deter e-cigarette use, highlighting enforcement challenges in controlling their extent in Thailand.

E-cigarette users in this study primarily accessed information through social networks, similar to findings in Jordan and Thailand, where social media influenced product orders and spurred curiosity about e-cigarettes. Studies in the US also link social media use to increased e-cigarette usage due to perceived advertising^32^. Conversely, non-users were less likely to seek information about e-cigarettes, potentially leaving them susceptible to inaccurate propaganda distributed through social media, which could spark interest in trying e-cigarettes.

Perceptions of e-cigarette health effects varied between smokers and non-smokers, with users generally holding lower perceptions of health risks compared to non-users, consistent with prior research^25^. These perceptions were associated with increased e-cigarette usage, as users often sought product-related information first, raising concerns about rising consumption without clear regulations. Additionally, beliefs about e-cigarettes’ effectiveness in smoking cessation influenced use intentions, aligning with previous studies, although long-term safety outcomes remained uncertain^15^.

Based on our findings, we recommend government policies for accurate e-cigarette information dissemination, focusing on health hazards for both smokers and non-smokers. Government-led education on e-cigarette products and their role in smoking cessation is essential. Additionally, monitoring online media for accurate e-cigarette information is crucial. To reduce e-cigarette use and stop future trends of increased usage, healthcare professionals in clinical settings should give patients accurate information on e-cigarettes. This study collected data from the general public nationwide, not only e-cigarette users, to provide insights representative of real-world perspectives.

### Limitations

However, there are limitations to consider. Conducting an online survey may have biased the sample towards smartphone users, potentially comprising predominantly health-literate undergraduates with possibly skewed perceptions of e-cigarettes, which may not be generalizable to the broader population in Thailand. Therefore, careful interpretation of the findings is advised. Additionally, the cross-sectional study design poses inherent limitations, including the inability to establish causality. An additional important weakness was the effects of the COVID-19 pandemic, which were not evaluated. To supplement the quantitative analysis, future studies should include qualitative studies and diversify the sample groups, especially youth under 18 years.

## CONCLUSIONS

This study revealed that the public can access information on e-cigarettes from people in their close surroundings and on social media. The perception of information about e-cigarette products and male gender were associated with a higher likelihood of being an e-cigarette user. To restrict the dissemination of e-cigarettes, the government should implement procedures to remain vigilant about the information provided about them on social media.

## Data Availability

The data supporting this research are available from the authors on reasonable request.
